# The Week's Work

**Published:** 1910-03-05

**Authors:** 


### The Week's Work.

### THE LONDON COUNTY COUNCIL AND AMBULANCES.

With a view to secure the prompt enforcement of the Metropolitan Ambulances Act, 1909, a letter has been addressed to every candidate at the ensuing County Council election by the Founder of the Street Ambulance Service, countersigned by the Honorary Secretary to the Bischoffsheim Ambulances, pointing out that it is not a question of party but of humanity to support the immediate establishment of an efficient ambulance service for London without delay. The candidates whose addresses could be traced consist of 114 Moderates and 111 Progressives, or together 225. Of these, 71 Moderates and 71 Progressives, or together 142 candidates, have replied, of whom 57 Moderates and 69 Progressives declare themselves in favour of the County Council establishing an efficient ambulance service in London similar to that now working in the City under the powers conferred by the Metropolitan Ambulances Act, 1909. Several of the other signatories undertake to give favourable consideration to the matter, and others decline to pledge themselves that the County Council Ambulance shall be similar to that now working in the City. We hope that the electors will stir up the 40 Progressives and the 44 Moderates who appear to take so little interest in the needs of London's citizens that they have not even troubled to return the postcard of inquiry. In any case, it is not thinkable that Greater London can longer be left without an adequate, up-to-date, and efficient Ambulance Service.

### AN AMBULANCE SERVICE FOR GREATER LONDON

The efficient working of the system of electromotor ambulances now organised in the City makes the absence of similar provision in Greater London more marked than ever. Under the Metropolitan Ambulances Act, 1909, full powers are given to the London County Council to establish and maintain such an ambulance service. For sixteen years, owing to the liberality of the late Mr. Bischoffsheim and the zeal and energy of Mr. Thomas Ryan, the street ambulance system has materially reduced the risks and sufferings of those who meet with accidents in the streets of London. Fuller facilities are, however, now called for, and the electors should make it their business to obtain pledges from the candidates who are seeking election to the County Council throughout the metropolis. The street accidents, which number many thousands annually, have increased by more than 60 per cent. within the last three years, and the County Council must perfect the ambulance organisation and so prevent the large amount of unnecessary suffering which at present exists.

### PATIENTS TO BUILD AND EQUIP A CHAPEL.

The Frimley Sanatorium is so excellently managed, and the work it does has been so successful, that anything connected with it must have a special interest for all who work among the sick. Equipped as it is with every facility for the scientific and special treatment of tuberculosis in its early stages, organised with an efficiency which no other sanatorium can probably claim, having secured results which are as remarkable as they are satisfactory, it now puts forward a novel scheme and invites the aid of the public. Lord Cheylesmore, the courteous and able chairman of the Brompton Hospital, who is chairman of its committee, explains that there is need for a chapel for the patients at the Sanatorium at Frimley. Last summer a memorial was presented to the committee by the patients at the Sanatorium expressing their earnest desire to be allowed to build and equip a chapel by their own labour as part of their treatment in physical work. This memorial led to the preparation of plans for the erection of a chapel by the architect who built the Sanatorium. The building has been designed with a view to simplicity, but is in keeping with the rest of the buildings, and the patients can undertake the greater part of the work. The estimated cost in the special circumstances is £2,000 and £500 more will cover the cost of equipment and skilled labour. It is noteworthy that the patients at Frimley have already constructed a concrete reservoir to hold half a million gallons of roof and surface water for use in the baths and as a storage in case of fire. They have also kept the Sanatorium in decorative repair and carried out other works. We hope Lord Cheylesmore will speedily receive the £2,500 asked for, and that the patients' chapel at Frimley will soon be an accomplished fact.

### ORGANISED CHARITY.

The meeting of the Social Welfare Association at the Mansion House on Friday, which was summoned by a letter signed by the Lord Mayor and the Chairman of the London County Council, has excited some interest. It is not easy to understand clearly the aims and objects of this association from the statement and documents which have so far been published. We learn, however, that it is not promoted by the Charity Organisation Society, though Mr. C. S. Loch, the well-known social economist, is actively interesting himself in its organisation. It appears that there are a number of Social Welfare Associations which have been started in the provinces, and that the object of Friday's meeting was to try and get a central body in London which will be able to induce the various local Social Welfare Associations to work upon the same lines and accept the same principles of administration. In other words, one chief object of the new association will be to remove the great incentive to loafing and begging which the overlapping of charity at present provides. We understand the new association has the Association of Subscribers to Charities behind it, and that the former is in fact a fuller development of the aims of the latter body. The new association will not administer direct relief, but will endeavour to arouse the interest of the men and women who support charities to promote the welfare of the State and also of its poorer citizens. The new association is to establish a number of committees of local councils in each London borough, on which the churches, friendly societies, trade unions, and the promoters of the relief of extreme poverty will all be represented. We shall watch the progress of the new association with interest, and hope it may accomplish many things which have been attempted but have so far failed to be effectively enforced.

### REFRIGERATING APPARATUS FOR GENERAL WARDS.

In the *Zeitschrift für Krankenpflege*, Chief Engineer Venator, of Wiesbaden, discusses very fully the question of providing hospitals and institutions with up-to-date refrigerating plant, illustrating his paper with admirable photographs and plans of the cooling rooms and plant used in the best German hospitals. He finds the steam plant by far the cheapest method where the waste steam can be utilised for heating purposes as well, though for cleanliness and efficiency he strongly recommends the electric motor. In warm climates a good refrigerating plant is doubly valuable, since by its means it is possible to ensure an equable temperature in the wards by allowing the entering air to circulate over cooled brine pipes. Comparatively little use has been made of the various systems available for regulating the temperature in this fashion; for one reason, because none of them are perfect, while they are all expensive and wasteful. In fact it is desirable that engineers should endeavour to evolve a good system whereby, in warm weather, the wards, theatres, and day rooms can be kept at a moderate temperature without too much waste and too great expense.

### REFRIGERATING ROOMS.

The objections to applying the cooling systems to wards and living rooms, however, are not so great in the case of store or provision rooms or mortuaries. One of the most satisfactory systems is in use at the municipal hospital at Wiesbaden, where it has been found specially satisfactory in dealing with large milk supplies. A large ammonia condenser is used, and the milk, which is provided in a sterilised condition, is placed in open vessels in roomy refrigerators, wherein cooled brine or distilled water is allowed to circulate. The ammonia method is also excellent for mortuaries. At Guy's Hospital, if we believe rightly, such a system was installed some years ago in the mortuary, and has been found to work quite satisfactorily, there being comparatively little waste, while the system is economical and cleanly. Usually the mortuary is fitted with its own plant, a special room being added to hold motor, compressor, and condenser. In some of the largest of modern continental hospitals—notably the Virchow and the Nuremberg Hospital—there is a central refrigerating plant, leadings from which are available for the whole economic division. This centralisation, however, entails more initial expense, more detailed supervision, and costs more in upkeep, and for that reason Venator advises duplicating the plant and the use of smaller apparatus. Venator's article is interesting, also, because he is at some pains to deal with the hygienic aspect of lowering the temperature. There is doubtless some justice in his remarks that the systems for regulating the temperature in the wards have not been fairly tried, since many physicians "have manufactured the bogey of chilling effects upon patients who are exposed in summer time to a temperature lower than that which prevails in the open air outside the ward." This fear, as he shows, is quite needless, since it is not necessary to cool down the atmosphere in the wards to a marked degree, but merely to create an atmosphere which is of an equable temperature and which contains sufficient moisture to enable the body to get rid of its superfluous heat.

### HOSPITALS AND ADVERTISING.

One of the most important duties of hospital managers and secretaries is to advertise the needs and claims of their institutions. A good income and good advertising go hand in hand; indeed, there is no other way of raising an income; and unskilful advertisement is as serious an error in hospital management as any of the better suspected errors: extravagance, the building craze, or a reckless overdraft, for instance. Originality is the main thing, and originality is too often painfully lacking in charity advertisement. To quote an example of mismanaged advertising we have only to mention how quite recently a certain children's hospital was in desperate need, and called a meeting of local influential persons to help in raising funds. What was the result? The meeting repeated itself about once a week, the agonising statement of financial distress was raised again and again, but the only people who had the opportunity of being moved by it were members of the same audience which had heard it the week before. So badly was initiative lacking that when a certain great personage went out of her way to express her sympathy by a letter, thereby giving, one would fancy, a fairly broad hint of how the public might be won, this letter was apparently buried in the archives of the institution instead of being thundered from every housetop with fruitful results. Now this is not the way to raise money. The necessary advertisements should be used to help the institution to help itself, with the editor's co-operation. That co-operation is generously forthcoming when the ability of the managers of a charity is sufficient to produce interesting copy of the most telling kind.

### THE DRUG MARKET.

It is difficult to account for the extreme dullness which prevails in the drug market; each week an improvement is expected, but always postponed, and the orders which do come in are of an unimportant character. Possibly the unsettled political situation accounts to some extent for the unsatisfactory state of the drug market, but that alone is hardly sufficient explanation. At last week's public drug sales in Mincing Lane very little interest was taken in the catalogues, and the majority of the lots were bought in, but where sales were effected prices were maintained, with the exception of Buchu leaves, which were again slightly cheaper. Ipecacuanha and its preparations are still quoted at the lately-advanced rates, and cod liver oil is practically unchanged in its position. Salts of lithium are tending dearer.

